# Increasing cellular lifespan with a flow system in organotypic culture of the Laterodorsal Tegmentum (LDT)

**DOI:** 10.1038/s41598-018-37606-3

**Published:** 2019-02-06

**Authors:** César R. Romero-Leguizamón, Mohamed R. Elnagar, Uffe Kristiansen, Kristi A. Kohlmeier

**Affiliations:** 1 Department of Drug Design and Pharmacology, Faculty of Health and Medical Sciences, University of Copenhagen, Copenhagen, 2100 Denmark; 2 Department of Pharmacology and Toxicology, Faculty of Pharmacy, Al-Azhar University, Cairo, Egypt

## Abstract

Organotypic brain culture is an experimental tool widely used in neuroscience studies. One major drawback of this technique is reduced neuronal survival across time, which is likely exacerbated by the loss of blood flow. We have designed a novel, tube flow system, which is easily incorporated into the commonly-used, standard semi-permeable membrane culture methodology which has significantly enhanced neuronal survival in a brain stem nucleus involved in control of motivated and arousal states: the laterodorsal tegmental nucleus (LDT). Our automated system provides nutrients and removes waste in a comparatively aseptic environment, while preserving temperature, and oxygen levels. Using immunohistochemistry and electrophysiology, our system was found superior to standard techniques in preserving tissue quality and survival of LDT cells for up to 2 weeks. In summary, we provide evidence for the first time that the LDT can be preserved in organotypic slice culture, and further, our technical improvements of adding a flow system, which likely enhanced perfusion to the slice, were associated with enhanced neuronal survival. Our perfusion system is expected to facilitate organotypic experiments focused on chronic stimulations and multielectrode recordings in the LDT, as well as enhance neuronal survival in slice cultures originating from other brain regions.

## Introduction

Cell culture techniques that allow *in-vitro* investigations are essential tools for studying cellular processes, and have been used extensively, among other applications, in examination of neuropharmacological effects of stimulation of receptors by natural and synthetic compounds. Culturing neurons of the central nervous system (CNS) is relatively easy to do from embryonic^1^ or very young postnatal tissue^2^. However, isolated adult CNS neurons are difficult to grow in culture, presumably due to damage of axons/dendrites, tight adhesion of integrated synapses, presence of glial cells, and requirements of supportive trophic, and other environmental factors^3^.

Organotypic culture of brain slices is an *ex-vivo* technique, which can be used to promote long-term neuronal survival, thereby allowing mid- to long-term manipulations in a preparation in which the gross cytoarchitecture of neuronal cell groups remains preserved. This approach is excellent for conducting physiological, immunohistochemical and pharmacological studies on well-identified neurons and interacting cells populations following chronic exposure to stimuli. Culturing of entire brain slices has been shown to be an important step forward in simulating more *in-vivo*-like situations, especially in tissue of adult or old animals in which the brain is more developed and more susceptible to damage during the obtaining of the slices^4^. Nevertheless, it remains an issue that slices from younger animals generally show superior survival than those from older animals.

The technique of organotypic culturing of brain slices was first described in the late 1940s, in which slices were cultured on glass coverslips using the roller-tube method^5^. This opened the door for deeper investigative neurobiological studies and promoted the development of technical improvements which resulted in adequate neuronal survival, preservation of tissue architecture, as well as persistent neuronal interactions with other cell groups, such as glial cells, e.g. astrocytes^6^. Later, the method of culturing slices on a semi-porous or semi-permeable membrane, that created an air-medium interface was implemented which enhanced neuronal survival and the quality of the tissue^7^. The semi-porous method is commonly used in many laboratories today.

Numerous studies have reported that there are several factors which are important to monitor in order to optimise neuronal survival in slice culture over a longer period of time. One of these is a short duration of time from decapitation of the animal to the point at which the slices are created^8^. A second important factor is the thickness of the slices, ideally between 150 and 400 microns, and slices should be made with a high quality vibratome^9^. A third condition is utilization of an adequate culture medium that allows a high quantity of neuronal nutrients which includes an optimal oxygen level^10^. Fourth, maintenence of flattening and transparency of the slices as evident under macroscopic evaluation, is important, and the degree of these parameters is often used as an indicator of slice health^11^. Finally, as slices in organotypic culture do not have a circulatory system that facilitates the entry of nutrients and the exit of tissue waste^12^, perfusion systems must be utilised which allow regular change of the culture medium with a turnover time of at most 2 days.

The lack of internal perfusion of brain slices presents a challenge to maintaining tissue health which can be difficult to overcome when using organotypic slice cultures. Although vasculature is present in the slices, since capillaries can be maintained in the middle of the tissue, as they lack blood flow, these vessels exhibit no activity and generate a chemical substratum that can sometimes be harmful to cells, suggesting that flow is important. Further, it is important to maintain flow in the organotypic culture as it is known in cerebral slices that small blood capillaries continue to produce proangiogenic factors that with the passage of the days in culture, favor angiogenesis and revascularization which assists in maintaining the vitality of the tissue and cells^13,14^. To enhance perfusion in order to promote cell survival, different models have been used in organotypic tissues to establish flow in the vessels, or vascular recovery strategies have been implimented, such as microperfusion systems, which have proved to be successful even in slices with a great thickness^15^. However they are often complex systems to use, or prove to be too expensive. Therefore, it is relevant to be able to develop a simple and inexpensive system that can replicate to some extent the effects generated in the brain tissue by blood flow.

Many different regions of the mammalian brain have been cultured using the organotypic semi-porous approach, including the cerebral cortex, striatum, basal forebrain, thalamus, hypothalamus, mesencephalon, inferior colliculus, substantia nigra, cerebellum, spinal cord, retina, and cochlea^16^, with the hippocampus being the most studied area using this cultivation technique^17^. While organotypic culture of brain stem neurons has been performed, including within the locus coeruleus (LC) and dorsal raphe (DR)^18–23^, organotypic culture of neurons of the laterodorsal tegmentum (LDT), which is an area of the brain adjacent to the LC and DR, has never before been reported in the literature. The LDT is a heterogenous nucleus, comprised of cholinergic, glutamatergic and GABAergic neurons and together with the LC and DR serves as a fundamental player of the brain stem’s ascending reticular activation system, and is responsible for regulating wakefulness and sleep-wake transitions^24^. The LDT also plays a critical role in the arousal of motivated states, such as that experienced during drug seeking^25^.

Focus of studies within our lab has been on examination of neuronal effects within LDT brain slices of acute application of drugs which alter arousal state. However, we would like to be able to perform *in vitro* studies of chronic effects of drugs on LDT neurons which remain in a communicating matrix. Further, we would like to study effects on neurons which source from non embryonic tissue which is not very early in the postnatal period. Accordingly, we wished to develop an organotypic mouse LDT slice culture from mice a few weeks of age. As validated by use of electrophysiology and immunohistochemistry, we report here that by using the conventional semi-porous approach, we were able to create viable organotypic brain slices sourcing from mice 21–30 days of age. Further, we show inclusion of a novel, tube perfusion system which ensured constant flow of medium to the slices, enhanced tissue quality and extended the lifespan of LDT neurons over that achieved by conventional techniques.

## Results

**Figure 1:**
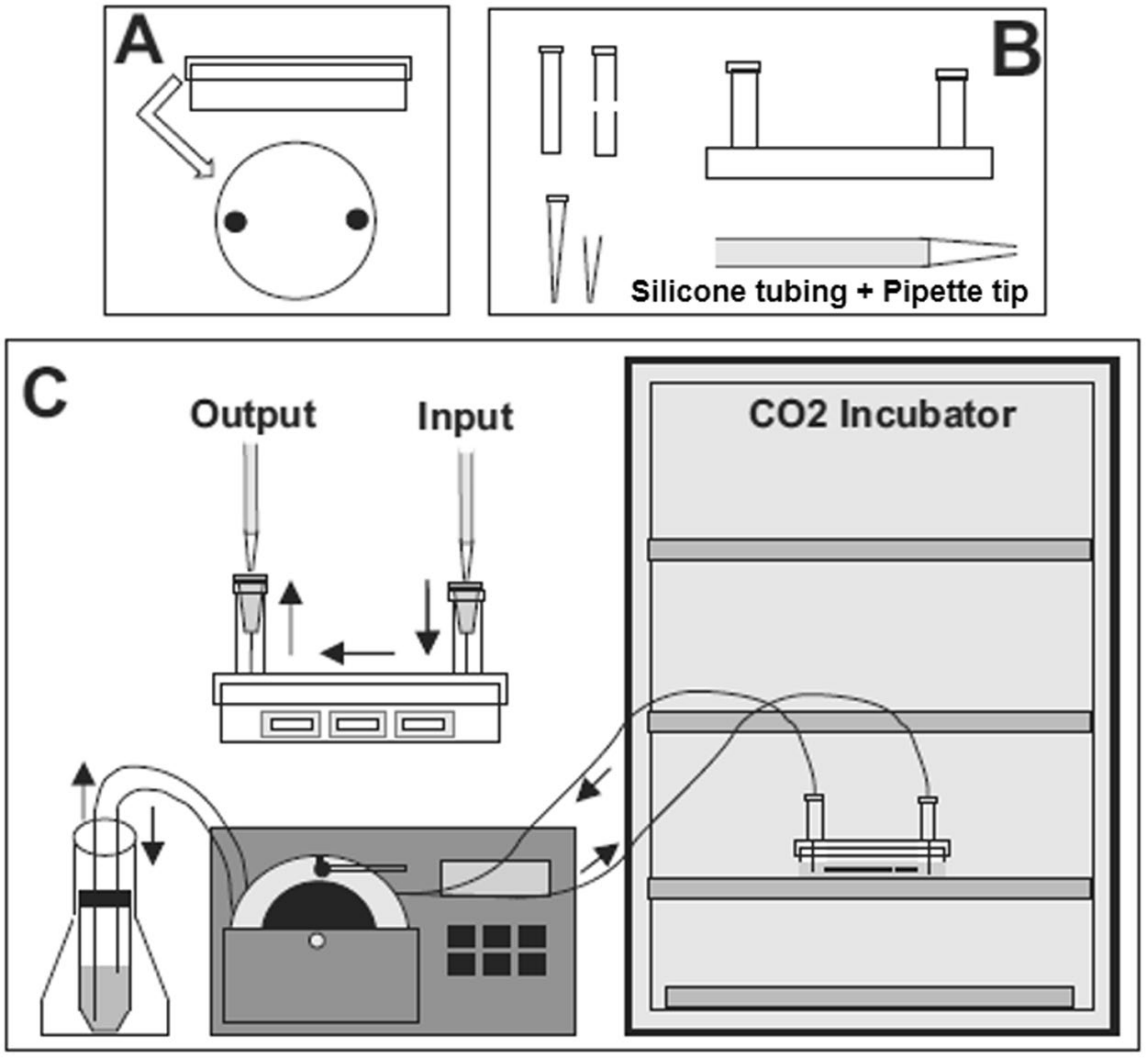
Scheme of the flow system. (**A**) Adaptation of the petri plastic box. (**B**) Elements that allow the coupling of the silicone tube to the petri dish. (**C**) Integration of the flow system to the conventional organotypic culture methodology. Our closed system allows media changes without opening the petri dish cover, reducing the probability of contamination in the slices. In addition, test drugs can be applied from the outside, further reducing contamination risk or abrupt temperature changes.

**Figure 2:**
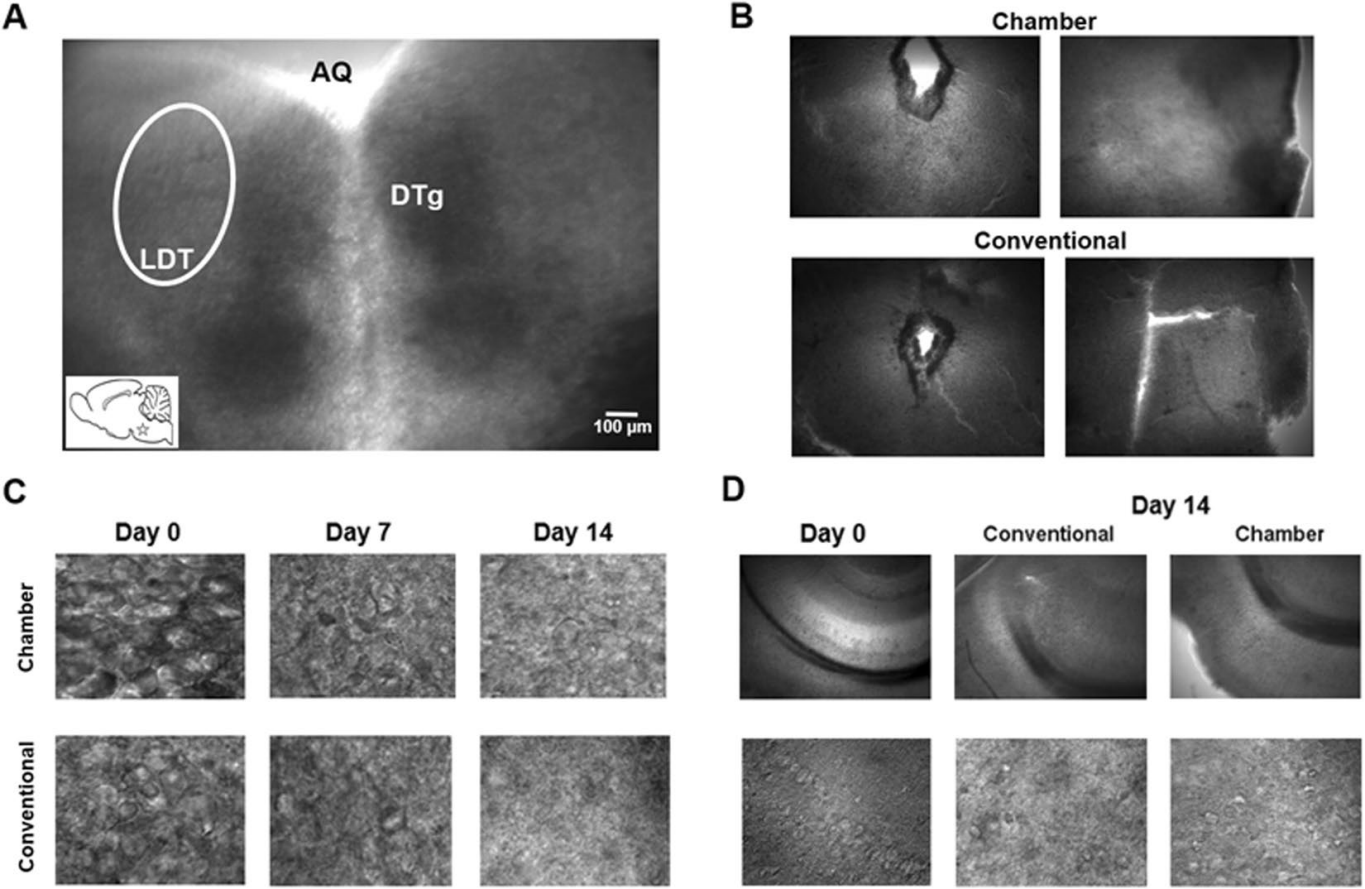
Microscopic evaluation of cell survival in LDT. (**A**) Coronal brain slice under low power optics (4X) in which the location of the LDT nucleus, indicated by a white oval, is seen relative to two anatomical landmarks, the aqueduct (AQ) and the dorsal tegmental nucleus of Gudden (DTg). In the lower left inset, the location of the LDT in the sagittal plane is shown. (**B**) Bright field images of LDT brain slices cultured in the flow system coupled with semi-permeable membranes or slices cultured using the conventional method with semi-permeable membranes alone, which show the difference in flattening of the cultured slices dependent on whether constant flow is present (Chamber) or absent (Conventional). (**C**) High magnification (40X) illuminated images of LDT cells taken across different days *in-vitro* (DIV) from slices housed in flow conditions or without constant flow in which it can be seen upon gross inspection that cell survival was superior in the slice group exposed to perfusion. (**D**) Comparison of the use of conventional methodology and the flow system in hippocampal slices. Visualization of CA1 in 4X (top panels) and 40X bottom, showing an enhanced cell survival after two weeks of culture with use of the flow system.

Organotypic cell cultures containing the LDT were made two different ways: by using conventional semi-permeable membrane methodology, and by adding onto the conventional methods a novel flow system that we have designated ‘chamber’ in figures, which was designed to enhance flow of nutritional solution to the LDT slice culture and optimize removal of waste (Fig. 1). For both approaches, the LDT was localised based on extensive experience with working in this nucleus, and by using a brain atlas^25^. The LDT was easily distinquished by first localising the dorsal tegmental nucleus, which appears darker under conventional brightfield microscopy. The LDT was located in a rough oval lateral to this nucleus (Fig. 2A).

### Vitality of Organotypic Brain Slice

In initial experiments, we noted that using the conventional system resulted in death in most of the cells and alteration in the appearance of the connective tissue. We repeated the procedure with the same result indicating it was endemic to our protocols, rather than due to a one-time error. Therefore, we altered the culture medium. Our second culture medium resulted in an improvement in cell survival and in the quality of the tissue. However, use of a third medium supplemented with horse serum, proved to be even more successful, supporting previous work. Further, we strived to reduce the duration of time from decapitation of the mouse to collection of slices to less than five minutes, which further improved cell survival. To determine the optimal time, cell death in the slices (n = 20) was evaluated by observation of apoptotic morphological characteristics of the cells after 12 hours of culture, dividing them in two equal (n = 10) groups according to the duration of time from decapitation to slices, group 1: 5–10 minutes, group 2: < 5 minutes. We noted a 60% decrease in apoptotic cells in the second group. Once this model was successfully established, our novel flow system was coupled to evaluate its impact.

Our novel perfusion system consisted of silicone tubes which were connected to a petri dish with polycarbonate cell culture inserts (Fig. 1). Following optimization of the correct length for the tubing, as well as the optimal pump speed, a proper level of medium for the brain slices was automatically maintained. The system was easy to use inside of a CO_2_ incubator, without causing leaks or changes in the temperature. Another distinct advantage was that it was easy to manipulate the adapter cover of the petri dish, allowing removal of selective slices without causing damage to the system or contamination of the medium, as contamination was not present in any of our tissue cultures.

Using conventional methods, after a few days (3–5), LDT slice cultures began to adhere to the semi-permeable membrane. However, in slices exposed to our novel flow chamber, slices began to attach to the membrane earlier (24–48 hours). Earlier adhesion was associated with a better quality of the tissue and cells, as those slices without flow lost their flattening and began to develop cracks, which was not seen to the same degree in slices exposed to the novel flow system. Macroscopic and microscopic evaluation revealed an unspecific cellular proliferation in the free edges of the slices, which with the passing of the hours confered a gelatinous aspect to the tissue. This process was very slow and was almost non-existent in the slices housed in the flow chamber (Fig. 2B). Additional comparisons of the cells of the LDT slice cultures revealed more robust persistence of cells using the flow system evaluated on the seventh day in culture when compared to the conventional method and this condition remained until day 14 (Fig. 2C). The flow system allowed neuronal survival up to two weeks *in-vitro* (DIV) with a peak in tissue and cellular quality around 7 DIV. For comparision, hippocampal slices were observed after two weeks of culture, which indicated that the flow system also enhances cell survival in other brain regions (Fig. 2D).

#### Cell Viability

Cell survival was evaluated and quantified by comparing the two slice culture methods. To this end, we utilized the MTT cell viability assay, which detects differences in metabolic activity by comparing measures of optical density. For this evaluation, slices (n = 62) were classified into 4 groups: A. Second medium and no flow, B. third medium and no flow, C. Third medium plus flow. Slices across the three groups were collected from mice who were between 21 to 30 days of age. A fourth group was also included (D) which was exposed to the third medium with flow in slices collected from mice within a younger range of ages (between 7–12 days of age).

**Figure 3:**
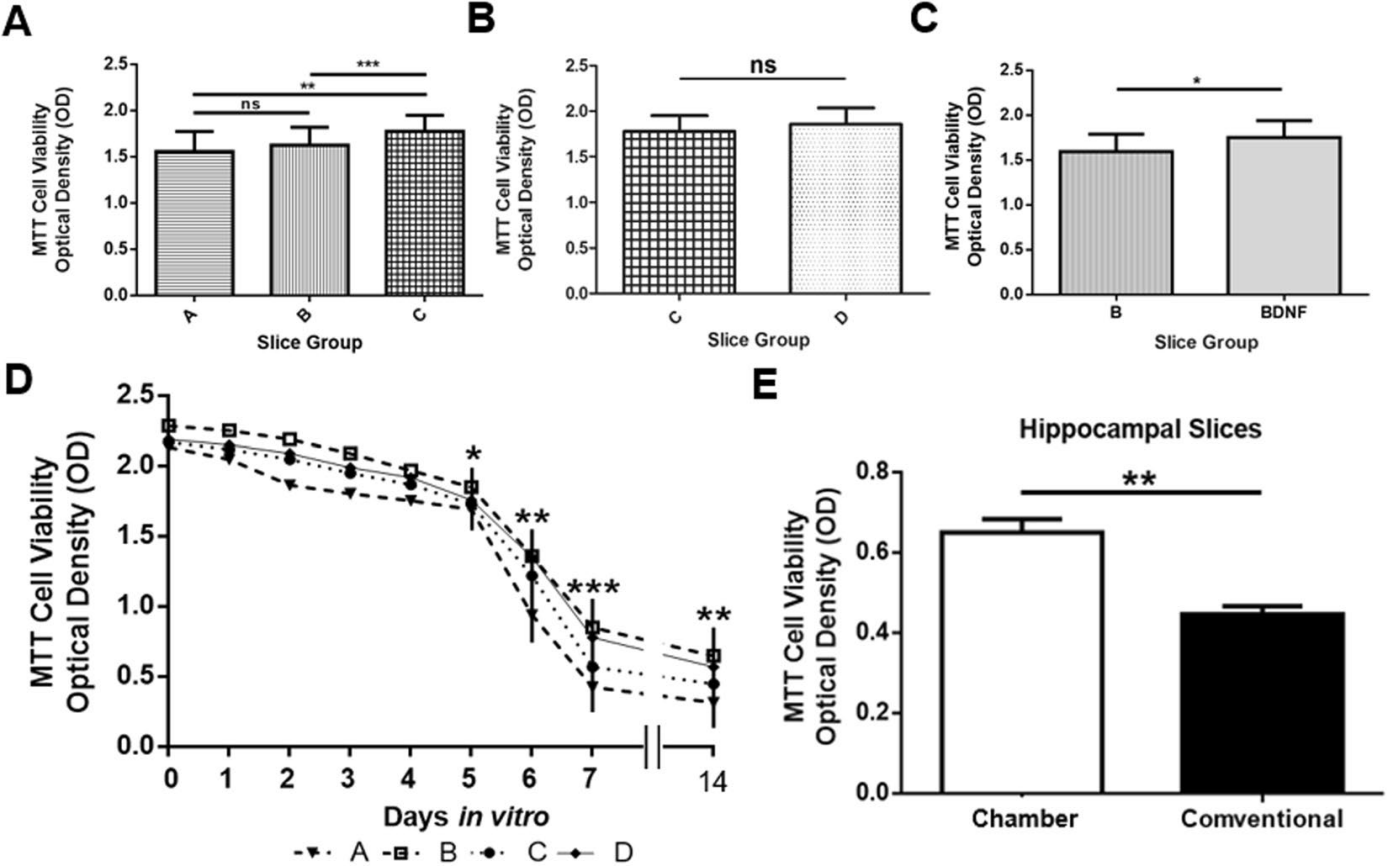
Cell viability as evaluated by the MTT assay. Groups: A. Second medium and no flow, B. third medium and no flow, C. Third medium plus flow (A-C in animals 21–30 days old), D. Third medium plus flow in younger animals (in animals 7–12 days old). (**A**) Comparison of the conventional methodology, the change in the culture medium and the addition of the flow system (**p = 0.002, one way ANOVA, and by Tukey’s multiple comparisons test significant difference between A –C** and B –C***). (**B**) No statistically significant difference when using the flow system in older (Group C) or younger animals (Group D) was detected. (**C**) The addition of brain-derived neurotrophic factor (BDNF) to the third culture medium was found to impart a significant difference in cell viability at DIV 7 (*p = 0.0200, paired *t-*test). Therefore, viability studies of cells at DIV 14 cultured with the chamber or the conventional system were all conducted with presence of BDNF in the media (**D**) Progression of cellular survival in relation to the days of culture. Data displayed as mean ± S.E.M. (*p = 0.03, **p = 0.002, ***p = 0.0003, one way ANOVA). (**E**) Cell survival in cultured hippocampal slices (n = 6; P14) with the conventional methodology and the flow system (**p = 0.0012, paired t-test).

Optical Density (OD) measures were greater with use of the chamber which ensured continuous flow into the culture plate (p = 0.002; ANOVA one-way and Tukey’s multiple comparisons test) (Fig. 3A). Interestingly, when the flow system was utilized, there were no statistically significant differences noted in optical density measures collected from mice at P21 to P30 (Group C) and slices from 7 to 12 day old mice (Group D), which suggests that the flow system can overcome well-known challenges faced by viability of cells in slice culture of tissue from older animals (Fig. 3B). Significant cell survival was detected after 5 days (*in-vitro* (DIV) in the cultures exposed to the flow system (p = 0.002; one way ANOVA) (Fig. 3D). When examined at 2 weeks DIV, hippocampal slice cultures also showed a significant difference (t-test p = 0.0012) (Fig. 3E).

**Figure 4:**
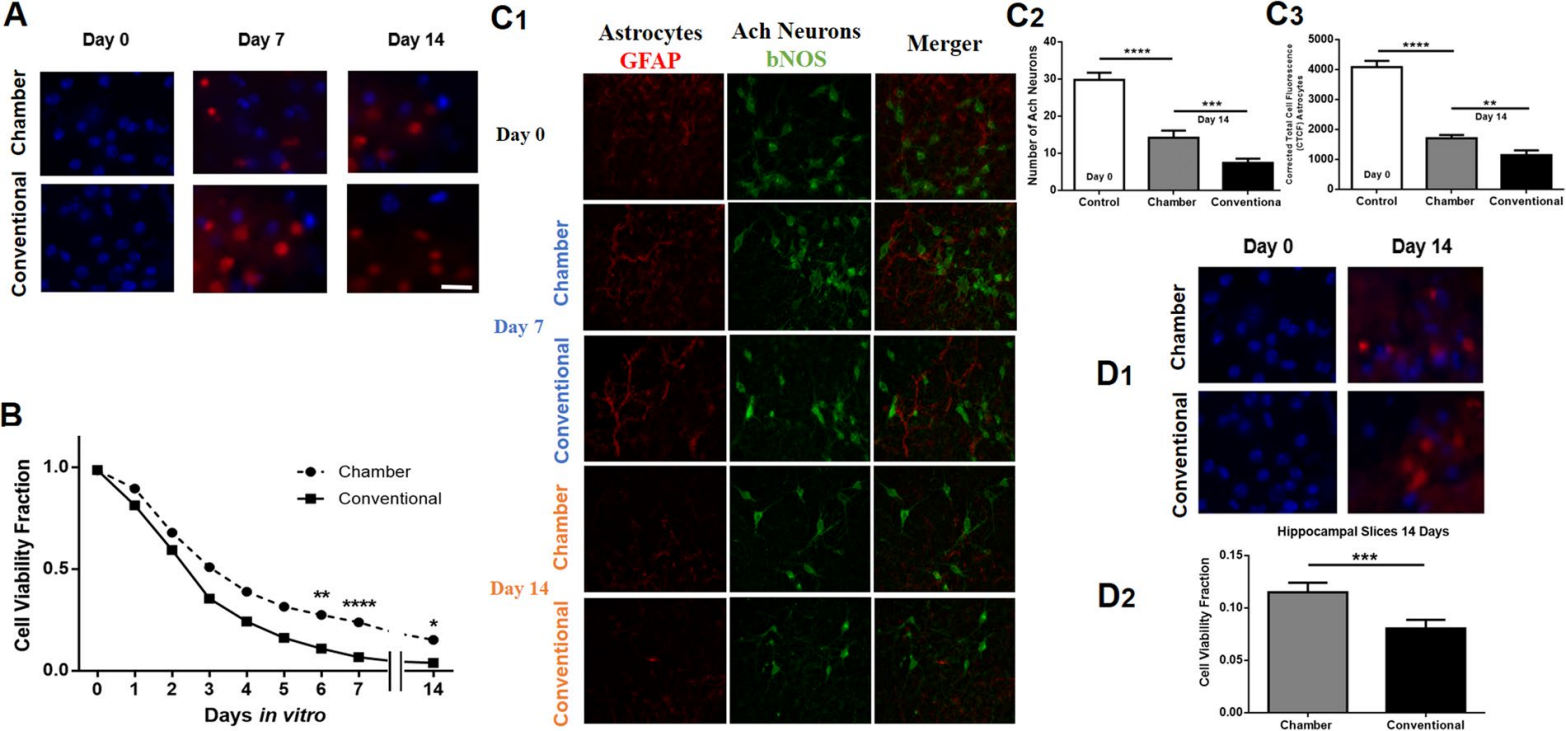
Fluorescence markers for cell survival in LDT. (**A**) Monitoring of cell survival with DAPI (blue) and PI (red) in sections of the cultured slices with and without flow system at DIV 0, 7 and 14 (Scale bar = 70 µm). (**B**) Cell fractions showing neuronal viability in the cultured slices with and without flow system during the days of culture (*p = 0.020, ****p = 0.0001, two way ANOVA), indicating statistical differences in this fraction after the first 5 days. (**C1**) Immunohistochemical evaluation of cholinergic neurons with anti-bNOS antibody (green), and astrocytes with anti-GFAP antibody (red). The survival of these cells is evident during two weeks of culture, with a greater impact on those cultured with the flow system. (**C2**) Comparison of the number of cholinergic neurons (ACh; bNOS positive) (****p = 0.0001, ***p = 0.0018, two way ANOVA). (**C3**) Immunoreactivity measure of astrocytes, with significant differences in the corrected total cell fluorescence (CTCF) (****p = 0.0001, **p = 0.0025, two way ANOVA) on day 14 with and without the flow system. (**D1**) Evaluation of cell survival in hippocampal slices cultured for 14 days with and without flow system. The DAPI/PI fraction indicates a lower number of surviving cells with use of the conventional methodology. (**D2**) Bar graph of statistically significant difference between the two culture methodologies in hippocampal slices (***p = 0.0012, paired t*-*test).

In the second evaluation, fluorescent markers were used to assess cell viability. DNA specific fluorescent markers, DAPI and propidium iodide (PI) were used to evaluate cell survival. Using these markers, slices were evaluated immediately after being obtained (Day 0) with subsequent repeat monitoring, up to 14 days of culture (Fig. 4A). Cell viability fractions were obtained from the relationship between the number of living cells (DAPI positive, PI negative) to the total number of cells (DAPI positive, PI positive). In the first 24 hours of culture, no differences were found in the average of cell survival between the conventional method (74.8%) and the flow system (81.1%). Nevertheless, after six days in culture a significant difference in the live/total cell ratio was found (48.59%, p = 0.0012, two way ANOVA), reaching a maximum difference in 7 DIV (71.91%; p = 0.0001; two way ANOVA) which was also present at P14 (36.93%; p = 0.025; Two-way ANOVA) (Fig. 4B).

Cholinergic neurons and astrocytes were identified with anti-bNOS and anti-GFAP antibodies, respectively, tagged with a fluorescent secondary (n = 40 slices). On day 14, a statistically significant difference was present in numbers of cholinergic neurons (p = 0.0001, two way ANOVA) (Fig. 4C1,2), as well as in immunoreactive measures (p = 0.0001, two way ANOVA) of astrocytes between the conventional and flow systems (Fig. 4C3). In parallel, immunoreactive measures were obtained from hippocampal slices showing a better cell survival with the use of the flow system (p = 0.0002: paired t-test) (Fig. 4D).

#### Electrophysiological evaluation of cell viability organotypic slices in the flow system

Our final comparison of cell viability of neurons in slices cultured with and without our novel flow system was conducted using electrophysiological, whole cell recordings in voltage and current clamp mode. Achieving a gigaseal between the cell membrane and the patch pipette is paramount to obtaining stable, whole cell recordings. In our first attempts to patch cells from both systems in slices up to 7 DIV, gigaseals were difficult to obtain, likely due to fragility of the cell membrane. Additionally, the current required to hold the cell at −60 mV often exceeded 100 pA, which is much greater than the often used criteria of definition of an acceptable whole-cell recording as that requiring holding currents less than 50 pA. We found in the second series of recordings which focused on 14 DIV, that gigaseals were easier to obtain, and low holding currents were possible, suggesting that inclusion of BDNF and/or the passage of time facilitated cell membrane health.

**Figure 5:**
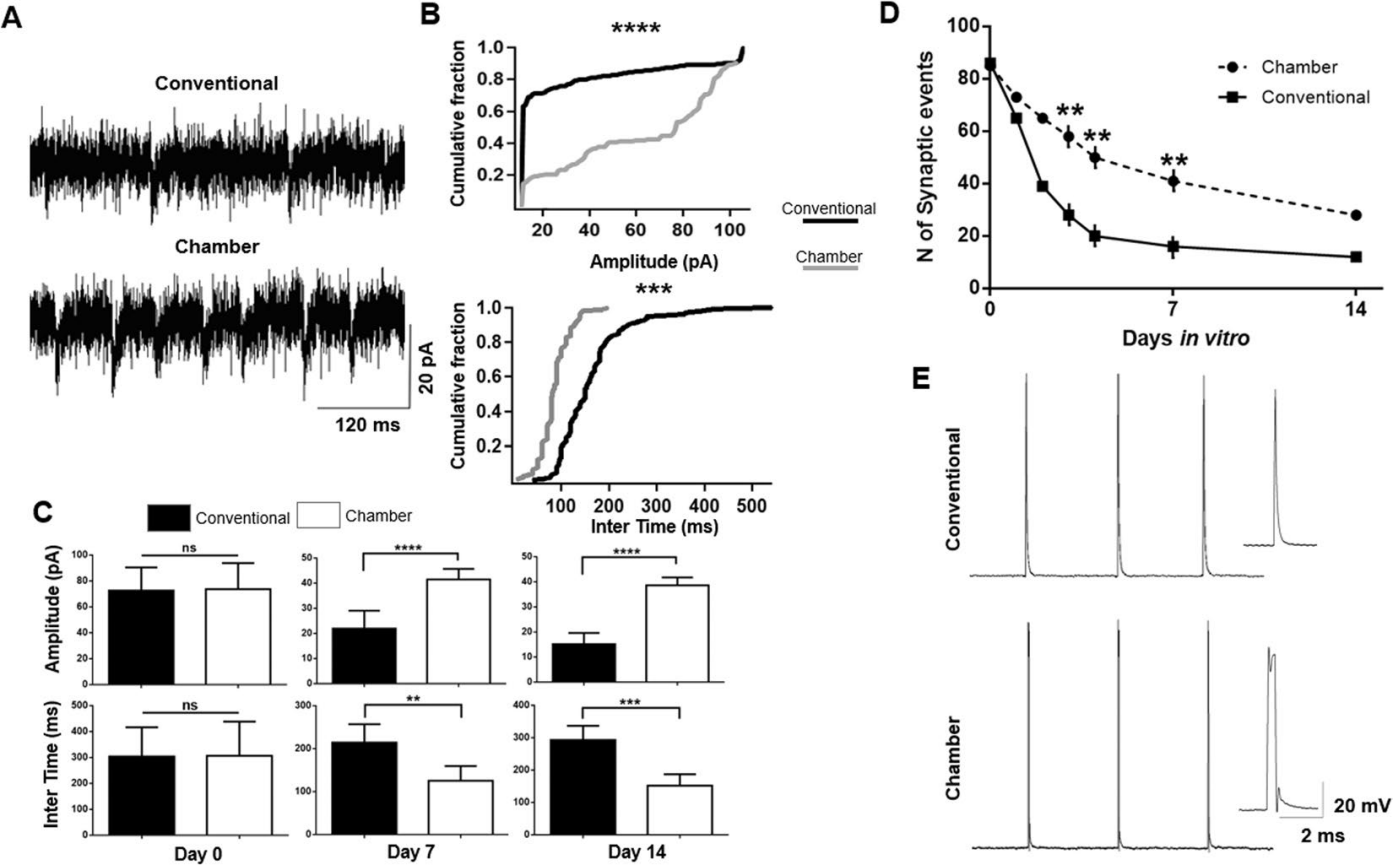
Assay of cell viability using electrophysiological recordings. (**A,D**) Recordings of membrane holding currents revealed that spontaneous excitatory synaptic potentials (sEPSCs) were present in cells cultured using the conventional and perfusion method. However, there was a significantly greater frequency of sEPSCs as well as a greater sEPSC amplitude of events from cells in the cultured slices exposed to flow. (**B**) Cumulative fractions reveal larger amplitudes of sEPSCs, as well as smaller sEPSC intervals from cells recorded in slices from chamber, as illustrated in two representative cells at day 14 (***p = 0.0015, ****p = 0.0001, Kolmogorov–Smirnov test). (**C**) Histograms of the average intervals and amplitudes of sEPSCs across the population of cells at 0, 7 and 14 DIV reveals a significant difference in these parameters dependent on presence or absence of flow. (**D**) Graph showing the average number of events in the slices with and without flow across several DIV days, showing a lower number of events in slices which were not exposed to the flow system (**p = 0.0016, two way ANOVA). (**E**) Generation of action potentials in the presence of a stimulus in current clamp mode was possible in slices cultured with flow (bottom panel).

Nevertheless, in voltage clamp mode, spontaneous postsynaptic membrane excitatory events (sEPSCs) could be detected in cells in culture (n = 56) with significant differences detected in frequency and amplitude at days 0, 7 and 14 (Fig. 5A). Cumulative fractions from cells on day 14 showed a significantly greater amplitude of sEPSCs, as well as a smaller interval of excitatory events, in the slice cultured with the flow system when compared to those in the slice maintained using the conventional methodology (Fig. 5B). Across days 0, 7 and 14, significantly shorter interevent intervals were obtained in cells recorded from slices exposed to the flow system (Average of intervals in 30 s samples of recording- Chamber: 142.6 ± 16.6 ms, n = 32; conventional: 221.12 ± 18.1 ms, n = 24; p = 0.0012, two tailed t-test). The number of events was significantly lower in cells cultured with the conventional methodology, compared to the cells cultured with the flow system (p = 0.0020; two way ANOVA) (Fig. 5D). The average amplitudes of sEPCSs were significantly greater in chamber slices, providing further evidence that cell health was promoted by the flow system (Chamber: 48.05 ± 1.6 pA, n = 32; conventional: 31.01 ± 2.8 pA; n = 24; p = 0.0018, two tailed t-test) (Fig. 5A,C).

In current clamp mode, by injecting brief depolarizing current pulses (800 pA; 5 ms duration), we were able to induce action potentials in 4 DIV LDT neurons cultured with the flow system, which was not possible when applying the same protocol to neurons cultured without flow (Fig. 5E). When taken together, our electrophysiological studies support the conclusion that the addition of our novel flow system to conventional semi-permeable membrane cell culture techniques improves cell viability in LDT brain stem neurons.

## Discussion

Our team has designed a flow system for the culture of organotypic mouse brain stem slices, which was shown in validation tests to improve cell survival for up to 14 days, according to those established by the cell survival tests (MTT, DAPI/PI), electrophysiology and immunohistochemistry. Our results indicated that the constant circulation of culture medium at a low speed was associated with a longer lifespan of cells of the brain stem in organotypic culture. Our findings are comparable to successful results of the microfluidic and microperfusion systems used in the cultivation of organotypic cortical and hippocampal slices^26,27^, as we see adequate cell survival for several days^28^, but our methodology offers the advantage of incorporation of a completely autonomous and easy to use system.

One of the major challenges to successful maintainence of neurons in organotypic slices is the loss of blood flow to the tissue. Neuronal cells have a very high metabolic rate with a large utilisation of glucose and a rapid production of waste. These factors make neurons highly susceptible to deleterious effects of a decreased blood flow, and there is an intimate relationship between neurons and the cerebrovascular system as optimal functioning of neurons relies on the vascular system in order to avoid tissue damage and neuronal loss^29^. Further, blood vessels present in tissue contain pro and anti-angiogenic growth factors that are important for revascularization of capillares in the brain slice^30^, as well as in *ex vivo* models of the blood brain barrier that use organotypic cultures^31^. Countering this consideration, while these factors may be beneficial, there are also factors produced in the slice, which need to be removed as they increase neural and glial mortality^32^. Accordingly, functional loss of the vascular system in organotypic brain culture presents a major hurdle to success in retention of live cells many days into the culture.

The LDT is highly vascularised both in humans and in rodents^33^ by small arteries which source from the midline basilar artery. Cholinergic neurons of the LDT contain structures known as end feet, which abut the blood vessels running through the LDT. While it is believed that the end feet allow hormones and NO which are produced by LDT neurons to be portaled to other brain regions, as well as that they allow neuronal sampling of factors in the blood such as those produced in stress or anxiety which can elicit responses^34^, the blood vessels also serve a role in providing nutritional support to cells in this nucleus.

While our data are the first known report of organotypic cell culture of neurons of the LDT, they are not the first to show organotypic survival of a cholinergic cell group without a flow system. Cholinergic neurons of the cortex were shown to survive in conventional organotypic culture^35^. With the addition of flow, we found that neuronal and astrocyte survival in the LDT slice culture was greater and, further, that no differences existed in tissue taken from young mice when compared to that in tissue taken from older mice. We interprete our findings to suggest that improved supply of nutrients and removal of waste facilitated tissue and neuronal lifespan in the LDT in culture in cholinergic neurons and astrocytes, as evidenced by immunohistochemistry. As ours is a closed system with solution acess independent of opening of the petri dish cover, potential for contamination with solution changes is lowered, temperature swings can be reduced, and possibilities exist to easily add test chemicals. Accordingly, our flow system could improve conditions for study of factors which control the LDT, and our enhancement could be useful for studies focused on characterizing the relationship between capillaries and these neurons and processes of angiogenesis.

While enhanced flow had a signficant effect on neuronal survival in the LDT, we detected that other technical factors could contribute to cell health, as has been noted by several others using organotypic culture^36^. We found that it was important to obtain the brain slices in the shortest possible time after decapitation, which is supported by literature^37^. In addition, there is likely an ideal range of slice thickness, as the more thick the slice, the less likely it is that adequate exchange of substance of the central tissue will occur, which will affect the survial of neurons, and this range could be region specific^38^. Different protocols have established that the optimal thickness for organotypic cerebral cultures is between 150 and 400 μM^9^. We did not systematically examine what the ideal slice thickness for the LDT is as the particular optical needs of our laboratory dictate a slice thickness no greater than 250 μM, and our desire to maintain connectivity limit how thin our slices can be. Regardless, when coupled with the flow system, slices of 250 μM demonstrated an enhance neuronal survival when compared to that achieved using conventional methods. However, it remains to be determined whether LDT slices of different thickness could prove to be associated with even greater survival when supported by our flow system.

Another important element to enhance neuronal survival in organotypic slice cultures is the composition of the culture medium. Although we did not perform an exhaustive study, but because it was the first time this region was cultured, and it is a very vascularized area which could have varying effects on culture, it was deemed prudent to explore different culture mediums. Our findings showed that the media supplemented with horse serum, BDNF, L-glutamine and D-glucose, resulted in the greatest neuronal survival in the LDT. Our data are supported by studies indicating that these components have a positive impact on the promotion of neuronal survival factors, as well as in enhancement of adherence of tissue in culture to the semi-permeable membrane which favors flattening^37,39^. However, some experimental protocols can dictate that a special medium must be provided. For example, working with very old animals can suggest a specific addition to enhance survivability or simulation of a specific pathological mode can guide media composition^40^. Interestingly, there is evidence of successful use of cerebrospinal fluid (CSF) as a replacement for regular culture media, which is appealing as this media would presumably be superior at providing a fuller range of endogenous factors available to normal tissue physiology^41^. However, replicability has been shown to be an issue and further, availability of this fluid for experiments presents technical barriers in rodents given the great difficulty of obtaining the CSF, and availability of human CSF is limited as sample collection necessitates bioethical considerations. Finally, when making medium changes every two or three days, there is a risk of removing too much or too little medium, altering osmolality or temperature too much, as well as promoting contamination. Therefore, some attention must be taken to the intervals in between media shifts, and it is possible that effects on this interval vary across different neuronal areas^42,43^.

Many different technical approachs have been used to establish or evaluate the number of live and dead cells in organotypic cultures and some of these can be specific to cellular subtype^44^. However, one of the most widely used methods for evaluation of cell viability in cultures is the MTT assay, which is also one of the most cost effective^16,45^. This assay relies on precipitation of the crystalline formazan in metabolically-active cells, which results in the conversion of the water-soluble, yellow dye into a dark purple (blue-magenta) color, that can be easily detected under appropriate optics. Other commonly used indicators of cell viability are the DNA-binding compounds, DAPI and PI, which are fluorescent markers that exhibit a high sensitivity in evaluation of CNS cell survival in fixed samples^16^. The results of these two methods which are widely used in organotypic cultures of brain slices support the same conclusion regarding the improvements of the flow system on enhancing cell survivability. We also evaluated our culture techniques using immunhistochemistry^46^, which allowed us to establish the positive impact on cell survival of cholinergic neurons and astrocytes of the LDT.

The electrophysiological evaluation of the viability of organotypic cultures has been widely utilized, especially in hippocampal slices^47^. Further, one of our primary goals was to use organotypic LDT slices to perform electrophysiological studies. Therefore, it was important for us to evaluate neuronal survival from the cultured slices using both the conventional and the flow systems. An electrophysiological indicator of damage is the absence and/or decrease in synaptic activity^48^. We were able to detect spontaneous synaptic activity using holding currents indicative of healthy patch conditions in neurons cultured from day 0 to 14 DIV from tissue cultured with both methods. LDT neurons of the brain slices that were cultured with the flow system did exhibit a greater number and amplitude of synaptic events as well as a significantly greater frequency of EPSC’s from 3 DIV, which indicates that the flow system is associated with a heightened maintenance of functional synaptic connections of LDT neurons^36,49^. We found that obtaining stable, whole cell recordings was easier from neurons in our population of 14 DIV tissue. This could be due to the inclusion of BDNF. However, while we did not explicitly examine this point, it could also be due to passage of time in culture. Other studies have shown that it can be necessary to maintain hippocampal tissue in culture for more than 2 weeks in order to recover from damage sufficiently for conducting electrophysiological recordings which could allow an adequate stabilization and maturation of intrinsic axonal projections^37,50^. Our study focused on 14 days of culture and we did not evaluate longer culture times. Therefore, while we do believe inclusion of BDNF did promote cell survival with either system, it remains a possibility that longer culture times promote LDT tissue stabilization, thereby facilitating high resistance seals, and cleaner break-ins, which would then require low holding currents.

Our data are the first to show that neurons of the LDT can be preserved in organotypic slice culture. Our results demonstrate that our flow system improved neuronal lifespan as well as the quality of the connective tissue in cultured LDT slices for up to two weeks. The LDT is a fundamental component of the ascending reticular activating system, which is critically involved in processes of arousal, attention, motivation and control of the sleep and wakefulness cycles^51,52^. Further, the LDT is one of the nuclei shown to degenerate in such CNS diseases as Parkinson’s and Alzheimer’s. Therefore, it is expected that organotypic slice cultures containing the LDT can be useful in chronic exposure studies addressing control of function of the matrix of LDT neurons which could be involved in processes of arousal and motivation, as well as in examination of cellular or network processes that can be involved in degeneration of cells in this nucleus. Further, our development of the addition of the easy-to implement, automated constant flow system to conventional semi-permeable slice culture methodologies is expected to be useful in improving the quality of neural tissue and neuronal lifespan in other regions of the brain, and further improvements are expected to facilitate longer cultivation periods.

## Methods

### Animals

NMRI wild-type mice (Charles River Laboratories, Germany) 7 to 30 postnatal days were used and housed under standard conditions (temperature 22–23 °C), on a 12:12 h light-dark cycle (Lights on/off at 7:00/19:00). Tap water and laboratory chow were available *ad libitum*. All experiments were permitted by the Danish Animal Welfare Committee, and complied with the European Communities Council Directive (2010/63/EU).

### Organotypic Brain Slices Preparation

Mice were decapitated following deep anesthesia with isofluran (Baxter, Unterschleibheim, Germany). The brain was gently removed and immersed immediately into ice-cold artificial cerebrospinal fluid (ACSF) containing in mM: NaCl 124; KCl 5; Na_2_HPO_4_ 1.12; CaCl_2_*2H_2_O 2.7; MgSO_4_ (anhydrous) 1.12; Dextrose 10; NaHCO_3_ 26; saturated with carbogen gas (5% CO_2_ + 95% O_2_). Coronal brain slices (250 µm) were obtained using a Leica vibratome (VT 1200S, Leica, Germany) which had been calibrated using Vibrocheck (Leica) to minimize tissue damage caused by vertical deflection. Following slicing, tissue was incubated in carbogen saturated ACSF for five minutes at 37 °C, then were carefully transferred to the *Brain Slices Flow Chamber*. Damaged slices were discarded.

### Culturing Media Exploration

Three different culture media were tested to find the one that could best preserve tissue quality and secure cell survival. First medium: 65% DMEM (Gibco), 25% Hank’s solution (Gibco), 6.5 mg/mL glucose (Merck, Germany), 10% Antibiotic-Antimycotic solution (Gibco) (penicillin 10,000 units/mL, streptomycin 10,000 µg/mL and Amphotericin B 25 µg/mL). In the second medium, Hank’s solution and glucose were replaced for 25% B-27™ Supplement (50X) (Gibco) and 6.5 mg/mL D-Glucose (Sigma-Aldrich) respectively.

The third medium was composed of 60% DMEM/F12 (Gibco), 30% Horse serum (Gibco/Lifetech), 10% Antibiotic-Antimycotic (Gibco), 6.5 mg/mL D-Glucose (Sigma-Aldrich), 50 µL/10 mL of L-Glutamine 200 mM (Sigma-Aldrich). In addition, effects of BDNF 250 ng/mL (Hellobio) were evaluated, and all experiments at DIV 14 included this factor. All media were filtered with disposable cellulose acetate syringe filter units, 3 mm Filter Diameter/0.20 μm Filter Pore Size (Micro Filtration Systems) and prepared under aseptic conditions in a laminar flow cabinet.

### Vitality Evaluation

MTT (0.5 mg/mL; Sigma-Aldrich) was added into the culture medium, by addition of 10% stock solution (5 mg/ml MTT in PBS; pH 7.4, Gibco). Slices were then incubated in a culturing incubator for four hours, washed twice with 1 mL PBS, and transferred to a solution made of 50% (v/v) N,N-dimethylformamid and 10% (w/v) SDS, pH 4.5. They were left in this solution 24 hours under constant stirring at 25 rpm (Heidolph Duomax 1030 Incubating Rocking Platform Shaker). Subsequently, the solution was centrifuged at 1900 rpm for 3 minutes. 150 µL of supernatant was used to record absorbance at 570 nm in a SpectraMax® Multi-Mode Microplate Reader M5 Fluorometer (Molecular Devices). Absorbance measurements were analyzed using SoftMax® Pro Microplate Data Acquisition and Analysis Software Version 5.4.1.

Immunohistochemestry was performed on slices incubated in BDNF-supplemented media either after slice removal from the flow chamber, culture plate or after electrophysiological experiments. These were placed in 4% paraformaldehyde overnight, then stored in 30% sucrose for 24 hours. Slices were re-sectioned to 40 µm (Leica CM 3050S) and then incubated with anti-bNOS (Rabbit polyclonal, cat #N7280, Sigma-Aldrich) followed by a fluorescent tagged secondary (goat anti-rabbit, alexa fluor 488 nm, A-11008, ThermoFisher) and anti-GFAP (Chicken polyclonal, ab4674, abcam) followed by goat, anti-chicken alexa fluor 594, (AB150172). Additionally, other slices were incubated for 3 periods of 5 minutes, in propodium iodide (PI, 1 µg/mL) (Sigma-Aldrich) and DAPI (1 µg/mL) (Sigma-Aldrich), pH 7,4.

Fluorescent signals were detected with appropriate Zeiss 59, fluorescent filter cube sets, DAPI & bNOS (358–463 nm)/PI & GFAP(472–578 nm), in an Axioskop 2, Zeiss microscope with a monochrome CCD camera (Axiocam MRM, Zeiss, Germany). Collected images (Axiovision 4,6 Zeiss) were analyzed using ImageJ software (National Institute of Health, Bethesda, MD).

### Electrophysiological recordings

Slices were placed in a recording chamber and continuously perfused with ACSF (37 °C) at a flow rate of 1 mL/min. For patch clamp recordings, pipettes were filled with a solution containing in mM: K-gluconate 144; KCl 2; HEPES 10; EGTA 0.2; Mg-ATP 5 and Na-GTP 0.3. Micropipettes were manufactured from thin wall borosilicate glass (outer dimeter 1.5 mm, WPI, USA) using a Sutter P-97 horizontal puller (Sutter Instruments, USA) with resistances between 5 and 9 MΩ. Using differential interference contrast optics on an upright microscope (Olympus BX50WI, Japan), slices were inspected to locate the region of the LDT with a 4X objective, then magnification was switched to 40X using a water immersion objective (NA 0.8, Olympus) to visualize individual LDT neurons suitable for patching. High resistance seals (>1 mΩ) were established between the patch pipette and the cell membrane, via an EPC9 patch-clamp amplifier (HEKA, Germany) in voltage clamp mode controlled by Pulse (HEKA; version 8.80). After that time, the membrane patch was ruptured and a holding current sufficient to clamp the membrane potential at −60 mV was applied. The holding current was sampled at a rate of 10 kHz using an Axon Instruments Digidata 1440A digitizer (Molecular Devices Corporation, USA) controlled by AxoScope 10.2 software (Molecular Devices Corporation). Recordings were considered unacceptable if the current necessary to hold the cell at −60 mV exceeded 50 pA. Evaluation of functional activity was performed by recording spontaneous miniature excitatory postsynaptic currents in LDT neurons, as well as by eliciting action potentials using current injections of 3 stimuli of 800 pA (5 ms), with an interval of 500 ms in current clamp mode.

### The Brain Slice Flow Chamber System

To improve cell survival, we developed a (tube) perfusion system, which was intended to replace blood flow and provide a constant supply of nutrients and clearance of cell debris. To achieve this, we created a flow system of culture medium through an adapted petri dish, as shown in Fig. 1. Petri dish adaptation. Under sterile conditions, two holes of 5 mm were drilled in the cover of a 60 × 15 mm Nunclon™ Delta Petri Dish (Thermo Fisher Scientific), each at opposite ends of the horizontal axis (Fig. 1A). The modified cover could be reused after sterilizing it overnight in ethanol 70% and exposing it for 30 min to UV light inside the hood. Connectors. With the help of a conventional blade, the distal (open) end of two plastic (needle) covers was cut to allow containment of two (sterile) hypodermic needles BD 27G X 3/4 inch 0.4 × 19 mm (Teruno-Agani), 22 mm and 20 mm in length. Plastic covers were glued (cyanoacrylate) to the Petri dish cover, over the holes but without obstructing them (Fig. 1B). Flow system. The “heart” of this system is a Peristaltic Multichannel Pump Type III (Ole Dich Instrumentmakers, Denmark). This pumped fresh culture medium from a 50 ml centrifuge holding tube (VWR) (which also has two 5 mm holes on the cover) to the modified petri dish via one channel, and at the same time, the action of the pump removed surplus culture medium via another channel, which resulted in maintainence of a steady level of medium in the dish. Two sections of 4 × 6 mm soft silicone translucent tubing (Length: 150 cm for output/125 cm for input. Ole Dich Instrumentmarkers APS, Denmark) were used for pumping and transport of the medium. Connecting the system: The petri dish was placed in a CO_2_ incubator with 5 or 7 Nunc™ Polycarbonate Cell Culture Inserts 10^8^ pores/cm^2^, Pore Size: 0.4 micron (Thermo Fisher Scientific) already inside the dish. Culture medium (2 mL) was added, while checking that the bottom of each filter was well soaked. On top of the covers, the two respective needles were inserted while verifying that one was submerged in the middle, and another on the surface, thus ensuring that medium level was always the same. To connect the needles with silicone tubing, it was necessary to use a pipet tip with FlexTop™ and UltraFine™ point 1–200 μL (VWR), cut at 28 mm and 25 mm (from proximal open). The silicone tubing was carefully attached to the incubator door’s threshold using adhesive tape. At the other end, the silicone tubes were inside a 50-mL tube, which was in contact with the medium and which was free of air. To prevent accidents and contamination, the tube was inside of a 250 mL Erlenmeyer flask covered with PARAFILM (Brand) (Fig. 1C). The ideal flow to maintain the system in equilibrium was around 100 μL/min, which was a rate which allowed the room temperature medium to warm up after entering the incubator and before contact with the petri dish.

### Data Analysis

Values are presented as mean values ± S.E.M. Statistical analysis were performed using an unpaired Student’s t-test, and one-way and two-way ANOVAs. Spontaneous synaptic events were detected and analyzed using MiniAnalysis (Synaptosoft, USA) in current recordings of 30 seconds epochs. Differences in cummulative distributions of the amplitude and inter event intervals of excitatory synaptic events were evaluated by the Kolmogorov-Smirnov test. Significance for all tests was set at p < 0.05. Figures were made in Igor Pro software (Wavemetrics, USA) and Prism Graphpad 6.0.

## Data Availability

The set of data analyzed and used for the present work will become available at The National Center for Biotechnology Information (NCBI) https://www.ncbi.nlm.nih.gov/.
